# Technological Overview of iPS Induction from Human Adult Somatic Cells

**DOI:** 10.2174/1566523211313020002

**Published:** 2013-04

**Authors:** Emilie Bayart, Odile Cohen-Haguenauer

**Affiliations:** 1 Laboratoire de Biologie & Pharmacologie Appliquée (LBPA) CliniGene, ENS – Cachan CNRS UMR 8113, 94235 Cachan, Paris, France;; 2 Department of Medical Oncology, Hopital Saint-Louis and Univ Paris-Diderot, PRES Sorbonne-Paris-Cité, 75475 Paris Cedex 10 France

**Keywords:** Human induced pluripotent stem cells, stem cells engineering, regenerative medicine, reprogramming methods, gene transfer systems, genetic instability.

## Abstract

The unlimited proliferation capacity of embryonic stem cells (ESCs) combined with their pluripotent differentiation potential in various lineages raised great interest in both the scientific community and the public at large with hope for future prospects of regenerative medicine. However, since ESCs are derived from human embryos, their use is associated with significant ethical issues preventing broad studies and therapeutic applications. To get around this bottleneck, Takahashi and Yamanaka have recently achieved the conversion of adult somatic cells into ES-like cells via the forced expression of four transcription factors: Oct3/4, Sox2, Klf4 and c-Myc. This first demonstration attracted public attention and opened a new field of stem cells research with both cognitive – such as disease modeling - and therapeutic prospects. This pioneer work just received the 2012 Nobel Prize in Physiology or Medicine. Many methods have been reported since 2006, for the generation of induced pluripotent stem (iPS) cells. Most strategies currently under use are based on gene delivery via gamma-retroviral or lentiviral vectors; some experiments have also been successful using plasmids or transposons-based systems and few with adenovirus. However, most experiments involve integration in the host cell genome with an identified risk for insertional mutagenesis and oncogenic transformation. To circumvent such risks which are deemed incompatible with therapeutic prospects, significant progress has been made with transgene-free reprogramming methods based on e.g.: sendaï virus or direct mRNA or protein delivery to achieve conversion of adult cells into iPS. In this review we aim to cover current knowledge relating to both delivery systems and combinations of inducing factors including chemicals which are used to generate human iPS cells. Finally, genetic instability resulting from the reprogramming process is also being considered as a safety bottleneck for future clinical translation and stem cell-therapy prospects based on iPS.

## INTRODUCTORY OUTLINE

In 2006, Takahashi and Yamanaka achieved the conversion of mouse fibroblasts into ES-like cells, almost indistinguishable from mouse ES cells, via the viral transduction of four transcription factors (Oct3/4, Sox2, Klf4 and c-Myc). They demonstrated shared characteristics with embryonic stem cells including the ability to form chimeric mice and contribute to the germ line. These studies opened a new field of stem cell research: Shinya Yamanaka obtained the 2012 Medicine Nobel prize in Physiology or Medicine, for pioneering this new field. Since these first demonstrations, many teams have succeeded in deriving iPS cells from human somatic cells. Significant progress has been made and many methods have been reported which may combine transcription factors and small chemicals. Modelling both monogenic and multigenic diseases is currently being pursued in many laboratories - including with the support and under the initiative from Big Pharma - as well as studies of complex genetic traits and allelic variation. Up until now, the most currently used strategy for iPS generation is gene-delivery via viral vectors systems. A summary of our current knowledge relating to both delivery systems and combinations of inducing factors used to generate human iPS cells is presented in this review. Strategies are also being considered which have been developed in order to circumvent vector integration-mediated risk for insertional mutagenesis and genetic instability, as major bottlenecks which may hamper further prospects of iPS-based derived therapies.

The lifespan of fully differentiated cells usually is short and they do not renew with few exceptions such as hepatocytes. Conversely, there is a pool of stem cells in tissues that holds extensive self-renewal capacity and is able to generate daughter cells which may further undergo differentiation into various lineages or terminally differentiate to reach a functional state. These adult stem cells (ASCs) can only generate a range of cell types specific to the tissue in which they reside and are thus called multipotent. In addition to ASCs, there are stem cells which hold an even broader differentiation potential, like the earliest possible, so-called embryonic stem cells (ESCs). ESCs can be isolated from the inner cell mass of the blastocyst before uterine implantation and maintained in culture without undergoing differentiation. They are able to generate all cell types of the embryo, but are not capable of initiating either the umbilical cord, trophoblasts or associated structures. These cells are described as being pluripotent. The successful derivation of human ESC lines extended their great potential to the study of human diseases and allowed to envisage the future prospect of regenerative medicine. However, this finding has also caused disquiet, as these cells were derived from *in vitro*-fertilised human embryos that in theory would have the potential to engineer a human being in full. However, besides significant ethical issues associated with the use of human embryos, this essentially is a limited source which, as such, also hinders broad therapeutic applications. A further disadvantage of ESCs is their unlimited proliferative capacity; this could cause tumour formation upon transplantation (so-called teratomas). Furthermore, ESCs would hardly be immune-compatible with a putative recipient patient, a feature which further restricts prospects for ESCs-based therapies. Several methods to generate patient-matched pluripotent cells have been developed, *e.g. *reprogramming through nuclear transfer to adult cells. Somatic cells could indeed be successfully reprogrammed to a pluripotent state by injecting the nucleus of an adult cell into an enucleated oocyte [1, 2] (reviewed in [3]). This leads to reprogramming of the somatic cell nucleus by the host cytoplasm. After several cell divisions, reprogrammed cells forms a blastocyst, which is at genetic match with the nuclear donor. Up to now, human somatic cell nuclear transfer, as it is called, is severely limited and is extremely demanding in terms of resources required. Also, the technique tends to cause some degree of cell damage and altogether is quite inefficient. As an alternative to oocytes, ESCs can be used for human somatic nuclei reprogramming [4]: this method also is rather inefficient and cannot be exploited for therapeutic applications given the resulting rate of tetraploid cells. Despite ethical and obvious technical limitations, somatic cell nuclear transfer clearly demonstrated that adult cells can be reprogrammed to a pluripotent state.

**Fig. (1):**
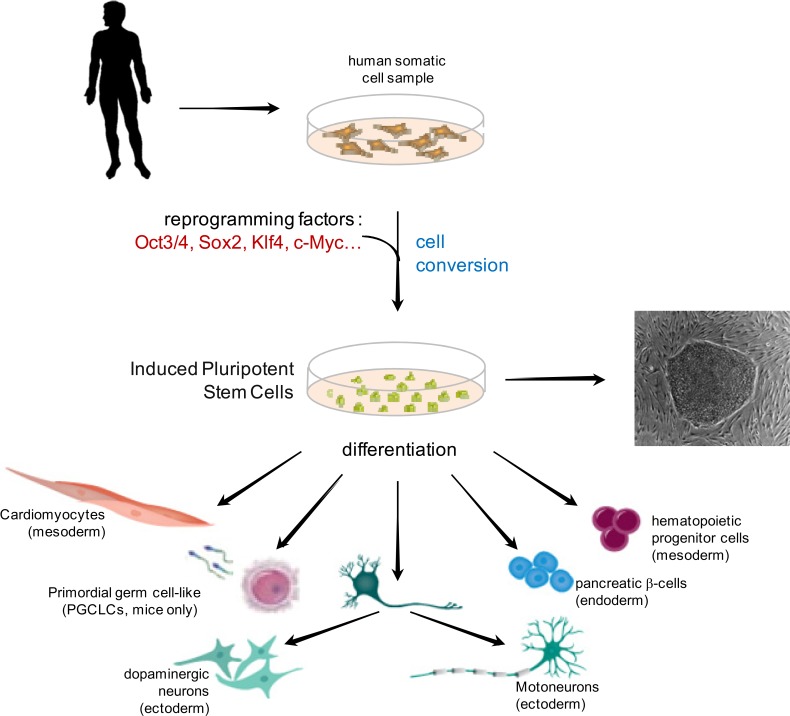
Schematic representation of adult somatic stem cells isolation and reprogramming into iPS pluripotent stem cells which in turn hold potential to re-differentiate into all three embryonic layers derived lineages.

In 2006, Takahashi and Yamanaka [5] first achieved the conversion of mouse fibroblasts into ES-like cells, almost indistinguishable from mouse ES cells in terms of pluripotency, *via *the viral transduction of four transcription factors (Oct3/4, Sox2, Klf4 and c-Myc). The latter were already identified as being involved in early embryonic development as well as cell proliferation and supposed to play a crucial role in ES cell identity [6-10]. They demonstrated the characteristics of embryonic stem cells including the ability to form chimeric mice and very recently it has been shown in mice that they can differentiate into primordial germ cell-like cells (PGCLCs) *in vitro* and matured into fully functional oocytes upon transplantation *in vivo* [11]. One year later, this team generated human iPS using the same strategy of forced expression based on four transcription factors [12] (Fig. **1**). This new field of stem cell research has attracted a great deal of public attention given the foreseen potential of induced pluripotent cells, derived from adult somatic cells. 

Since these first demonstrations, many teams have successfully derived iPS cells from human somatic cells. Significant progress has been made and many methods have been reported which may combine transcription factors [13] and small chemicals [14, 15]. Up until now, the most currently used strategy for iPS generation aiming at basic research is gene-delivery *via *vectors systems. Retrovirus, lentivirus, adenovirus and plasmid are the most widely used, although adenovirus has not been shown to work into human cells. Human iPS cells are relevant to a wide range of applications such as test substrates for drugs, evaluation of toxicity, differentiation, disease modeling and therapeutics screens. Modelling both monogenic and multigenic diseases is currently being pursued in many laboratories, including big pharma, as well as the study of complex genetic traits and allelic variation. iPS cells can indeed be generated from cells sampled from affected-patients [16] once the phenotypic expression of the disease has been well-characterized in them: such information is unknown when considering ES cells. A summary of the current knowledge relating to both delivery systems and combinations of inducing factors as well as chemicals used to generate human iPS cells is presented below. This review also includes transgene-free reprogramming approaches which have been developed in order to circumvent vector integration-mediated risk for insertional mutagenesis.

## 1 DELIVERY METHODS

**Fig. (2):**
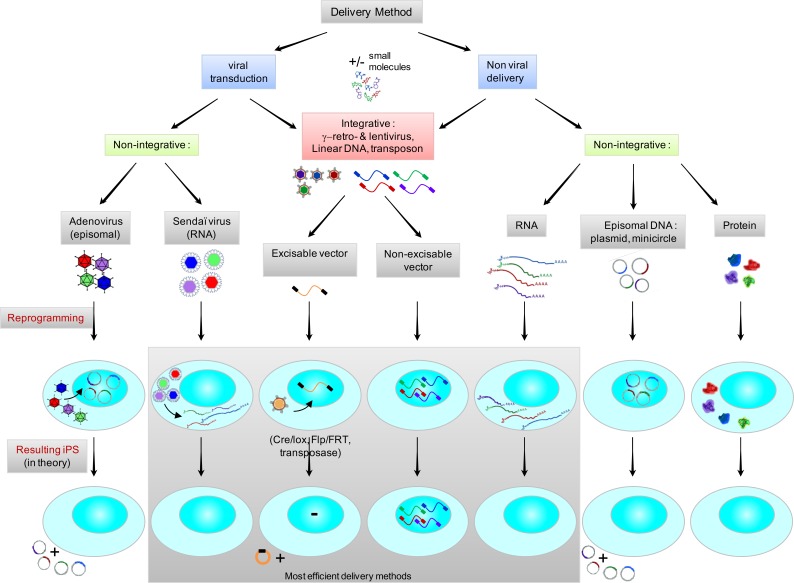
Schematic representation of technological options for iPS induction : viral, non-viral ; integrative, non-integrative, transgene-free and their resulting persistence or absence of genomic scars. The most efficient delivery methods are highlighted.

The reprogramming concept consists in the ectopic expression of a set of core pluripotency-related transcription factors in a somatic cell. In most cases OCT4, SOX2, KLF4 and MYC are used and represent the commonly so-called OSKM cocktail. If successful, tightly compact colonies growing in ESC culture conditions appear on the culture dish. These colonies are morphologically, molecularly and phenotypically related to ES cells. Since 2007 and the first generation of human iPS cells by Yamanaka’s team, more than 100 studies have been published which report on human iPS generation (For review see [17], http://intranet.cmrb.eu/reprogramming/home.html) which describes a number of studies published on mouse and human cells, among which some are pivotal (Fig. **2**; Table **1**).

### 1.1 Integrating Vectors

#### 1.1.1 Viral Integrating Vectors

In the original report of iPS induction, the delivery of pluripotency transcription factors was performed *via ***gammaretroviral** Mo-MLV (Moloney murine Leukemia Virus)-based vectors such as pMXs [12, 18] or pMSCV [16, 19, 20]. These vectors are replication-defective since regions encoding for the proteins necessary for additional rounds of virus replication and packaging are deleted from the viral genome. Defective gamma-retrovirus genomes have a cloning capacity up to 6-8kb, and are able to transduce target cells according to the envelope pseudotype under use. In actively dividing cells the efficiency of transgene delivery can reach up to 90%; a major limitation of this technology is that slowly- or non-dividing cells, such as neurons, are resistant to gamma-retrovirus-mediated transduction. It has been long identified that retrovirally shuttled transgenes are silenced in ES cells [21, 22], as well as in iPS cells [16, 23] through mechanisms involving methylation and epigenetic modifications [24]. In fact, transgene silencing is important since iPS cells are being considered as duly and fully reprogrammed only upon both up-regulation of endogenous pluripotency genes and down-regulation of the transgene expression [25, 26]. Despite practical advantages, gamma-retroviruses have been associated to major drawbacks in particular in clinical trials where insertional mutagenesis resulted in the development of malignancies. It thus became obvious that alternative approaches to retrovirus-mediated gene transfer should be considered especially when including a known oncogene like c-myc.

Unlike gamma-retrovectors, so far no malignancy resulting from insertional mutagenesis has been reported with **lentivectors**. These distinct subclasses of retrovirus vectors derive from either HIV-1, HIV-2 (human), SIV (simian) or EAIV (equine) and have been successfully used to generate iPS cells. A unique feature of lentiviruses is that they are able to transduce both non-dividing (slowly dividing or quiescent but metabolically active cells) and dividing cells, allowing the generation of iPS from most cell types. In addition, their cloning capacity is broader than that of gamma-retrovectors and they exhibit higher transduction efficiency, of human cells in particular. Like gamma-retrovectors, lentivectors are expected to be silenced during the reprogramming process. However, repression occurs to a lesser extent with lentivectors, a feature which in some instances may both prevent full reprogramming of cells [25] as well as indefinitely maintain unwanted expression of transcription factors and oncogenes used for reprogramming.

#### 1.1.2 Non-Viral Integrating Vectors 

An alternative to viral vectors is the standard DNA transfection of plasmid DNA *via *liposomes or electroporation. However, compared to viruses, transduction efficiency is extremely low which makes it unlikely that a single cell will indeed capture all reprogramming factors at once. A major improvement was introduced with the development of **polycistronic** vectors expressing all induction factors driven by the same promoter. In these constructs, each cDNA is separated by a self-deleting 2A peptide sequence from picornaviruses [27, 28] which allows ribosomes to continue translation of the second open reading frame (ORF) when the protein encoded by the previous one is released, so called the ribosomal skipping mecanism. Kaji *et al. *[29] were able to generate iPS from mouse cells and showed that a single copy of the polycistronic cassette was sufficient to achieve direct reprogramming.

There is no evidence that human iPS could be obtained after a single round of transfection using “conventional” induction factors. However, one team was able to isolate human iPS cells when a mi-RNA involved in epigenetic modification was used and antibiotics selection was applied for stable integration [30]. In most cases, non-viral vectors are now used as transient delivery systems which are further considered in section 1.3.2, below.

#### 1.1.3 Integrating Vectors and Insertional Mutagenesis

One major drawback of integrating delivery systems, whether viral or linear DNA vectors, towards induction of pluripotency is related to undesired transgene reactivation, a phenomenon which frequently occurs in differentiated cells derived from iPS, as this may lead to tumour formation resulting from *e.g. *over-expression of oncogene related factors such as c-*MYC*. Therefore, other transcription factors combinations have been investigated, which would exclude *c-MYC *and still allow full reprogramming [13, 23, 31, 32]. Another way to prevent re-expression of oncogene related factors is to control expression *via *a Tet-inducible system, which allows transgene repression in iPS like colony and further selection of fully reprogrammed cells [16, 33-37]. Authors confirm by Q-PCR that in iPS cells, ectopic transgenes used for reprogramming become inactivated while endogenous pluripotency regulators are reactivated. Buecker *et al.* 2010 [32], have demonstrated that constitutive activation of the reprogramming factors through an inducible system prevents iPS generation and maintains cells in a “poised-near-to-pluripotency” state where some endogenous pluripotency factors are activated whereas others are not, which harbour ambivalent histone status. These data confirm that iPS could be obtained only after removal of doxycycline so that the inducible transgenic reprogramming cassette is repressed to allow iPS formation. There is however no published information concerning putative re-expression of transgenic inducible reprogramming factors. Whether using an inducible reprogramming cassette is a safer option than a conventional one remains to be demonstrated.

In addition, iPS cell lines generated with integrative vectors carry randomly distributed transgenes insertions [38] that harbour the risk for potential insertional mutagenesis and subsequent development of malignancies when inserted nearby sensitive sequences. In fact, Kane *et al. *[39] have shown that iPS cells could be generated without transcription factors, in merely transducing human fibroblasts with lentivectors only expressing the green fluorescent protein (GFP), though at very high multiplicity of infection (MOI). Primary fibroblasts transduced at MOI 200 gave rise to iPS cells which contain as many as 20 integration sites. This study comes as a striking illustration of the extent of deregulation into which insertional mutagenesis may result, reminiscent of helper retrovirus pathology induced in rodent.

In fact, the use of polycistronic vectors considerably reduces vector copy number integration per cell, a feature which is expected to significantly decrease the risk for insertional mutagenesis. Based on the former observation that a single polycistronic cassette expressing all transgenes under the same promoter from linear DNA is able to allow full reprogramming of somatic cells, polycistronic gammaretroviral and lentiviral vectors have been developed which translate in the successful generation of human iPS cells [40, 41].

### 1.2 Excisable Integrating Vectors

#### 1.2.1 Viral-Derived Excisable Vectors and Heterologous Recombination System

As a next step towards safety improvement, excisable integrating vectors have been engineered in order to generate transgene-free iPS and help prevent above-mentioned drawbacks as well as the following. In addition to being placed under the control of viral promoters, the stable integration of transgenes encoding for transcription factors or oncogenes involved in cell proliferation such as *c-MYC, *harbours a substantial risk of malignant transformation should reprogramming factors not be fully silenced or incidentally be reactivated during differentiation. Moreover, viral promoter reactivation could lead to the deregulation of *cis*-neighbouring genes: the latter represents an additional mechanism which might compromise cell-cycle integrity. Excisable lentivirus vectors have been engineered which include both a ***loxP*** site in the 3’LTR and an inducible promoter driving transgene expression. During virus reverse-transcription, the *loxP *site is duplicated in the 5’LTR so that the integrated transgenic cassette is flanked with a *loxP *site at both ends. The excision of the reprogramming factors follows the targeted and transient expression of **Cre recombinase** in transduced cells which induces a recombination event between *loxP *sites. Using this system, Jaenisch and his group [42] were able to generate transgene-free human iPS cells which are able to maintain their pluripotent state and display a global gene expression profile similar to human ES cells (Fig. **2**)**.** These iPS cells could further differentiate into dopaminergic neurons [42]. The major limitation of this study is that reprogramming factors were primarily integrated at different independent sites which resulted in multiple transgenes excision upon Cre recombinase expression. In fact, multiple and simultaneous recombination reactions could lead to genome rearrangement and genomic instability. In order to overcome this drawback, polycistronic lentiviral vector encoding for defined reprogramming factors separated by 2A sequences resulting in the integration of a single reprogramming cassette floxed by two *loxP *sites have been designed [26, 43]. Following Cre recombinase mediated excision, the iPS cells lines generated harbour only three lentiviral LTR signatures which consist of a single *loxP *site that does not interrupt coding sequences, promoters or regulatory elements. Although conceptually elegant, this system holds a risk for non-specific recombination events and genomic instability should Cre recombinase expression not be tightly enough controlled.

Another commonly used heterologous recombination system is the **Flp/FRT recombinase/targets** system from *Saccharomyces cerevisiae *[44]. While it is supposedly less efficient than the *Cre/loxP *system [45] it conversely exhibits far less toxicity, a feature which is essential when working with primary cells [46]. To date, there has been no report of human iPS cells generation, while murine iPS cells have been generated using this system with a polycistronic lentivector in which the reprogramming cassette was flanked with two FRT sites. These mouse iPS cells were further transduced with empty MLV retrovirus-like-particles which shuttle the Flp recombinase fused to the Gag-pol polyprotein. This process resulted in the complete removal of the reprogramming cassette [47]. Transgene-free iPS resulting from heterologous recombination systems thus represent a more suitable source of cells towards human disease modelling. However, these iPS cells still harbour scars of insertion sites and are not “genetically clean” pluripotent stem cells, a feature which might still alleviate translation to cell-based therapies.

#### 1.2.2 Transposon-Derived Excisable Vectors

Besides viral derived systems, linear plasmids which encode a polycistronic cassette floxed with two *loxP *sites have also been tested for cell conversion [29]. While transgene free mouse iPS were generated, so far there is no evidence that human iPSCs could. In order to address the reprogramming ability of their non-viral single-vector system in human cells, Kaji and co-workers enhanced stable transfection efficiencies using a ***piggyback*** (*PB*) transposon-based delivery system which mediates genome integration at higher efficiency than would with linearized plasmids. Transposons are mobile genetic elements which can move from one position to another within the genome through an excision/insertion mechanism. As a vector system, *PB *transposon requires only 13 bp inverted terminal repeats (ITRs) and an active transposase, the enzyme which catalyses insertion and excision [48, 49]. The *PB *system is usually composed of a donor plasmid called transposon, which shuttles the transgenic sequence of interest flanked by the 5’ and 3’ ITRs. The latter is co-transfected with a transposase expressing helper plasmid that mediates integration [48-50]. Using these *PB*-based reprogramming vectors, both Kaji *et al. *[29] and Woltjen *et al.*[51], were able to generate human iPS cells from fibroblasts and subsequently delete the transgenes. In these studies, the authors demonstrated the traceless elimination of the reprogramming factors and scar-free excision of the inserted transposon without modifying the sequence of the integration site: this feature is unique to *PB*. Another transposon, the ***Sleeping Beauty*** (*SB*), was assembled in combining fragments of silent and defective Tc1*/mariner *elements from salmon fish [52]. The reconstructed *SB *showed the best transposition efficiency in vertebrate cells than any other transposon tested at that time. Most recently, a novel super-active transposase has been derived from *SB*: this **SB100X** mutant is a 100-fold more potent in HeLa cell lines compared to the originally resurrected *SB *[53]. Of note, it received the molecule of the year award in 2009. The efficiency of SB100X mediated transgene insertion is similar to viral transduction in generating both mouse and human iPS cells [54] but the integration/excision process is not entirely scar-free as with *PB*.

Both *piggyback *and *SB*-based system allow the removal of the reprogramming cassette and its site-specific exchange through a targeted recombination between the reprogramming cassette and a gene of interest, through the so-called Recombination-Mediated Cassette Exchange (RMCE) process. These features make the transposon/transposase system one of the best choices for delivering reprogramming factor into a broad range of somatic cells with view to generating “genetically clean” iPS cells [55]. However, transposon-based reprogramming is essentially depending on the delivery method which could represent a limitation when addressing some primary cells due to resistance or toxicity related to DNA transfection methods: lipofection, electroporation or nucleofection. In addition, it must be underlined that the transposition reaction in not always precise, as for instance Wang *et al. *[56] reported on alterations found in 5% of the transposition events. Moreover, the transposase promotes both deletion and integration at similar efficiencies, allowing the transposon to “jump” from site to site as long as the transposase is expressed: uncontrolled off-target repeated transposition could cause footprints and/or genetic rearrangement in the genome of human iPS cell generated. Therefore, the transposase expression window needs to be tightly controlled. Recently, Galla *et al.* [57] proposed an improved approach based on retrovirus particle-mediated mRNA transfer which allows transient and dose-controlled expression of SB100X. This was shown to both support efficient transposition and prevent related cytotoxicity. Although major improvements of both safety and quality of iPS cells are expected, the precise consequences of transposon-based system on the genomic stability of reprogrammed cells still need to be scrutinised and be it the case, ways of improvement sought.

### 1.3 Non-Integrating Vectors

#### 1.3.1 Integration-Free Viral Delivery

As persistent expression of reprogrammning factors should be avoided following iPSC generation, transient expression based on non-integrating vectors could help circumventing putative insertional mutagenesis. Along this line, **integration-defective retrovectors** have been engineered taking advantage of inactivating mutations introduced in the viral integrase. Integration-deficient gammaretroviral vectors have been described [58] which translate into very low titres. In addition to this bottleneck, their inability to transduce non-dividing cells makes it unlikely to fit the demands of most experiments. The so called IDLV-platform (Integration Deficient Lentivirus Vectors, for review see [59]) has attracted a lot of attention including with view to clinical translation in gene therapy settings. Therefore, like any episomal transgenic DNA IDLV may persist only transiently and be further diluted slowly with time and cell-divisions [60-62]. Surprisingly, so far, no iPS cells could be generated using integrase-defective lentivectors.

One of the first attempts to generate integration-free iPS cells was reported by Stadtfeld *et al. *[63], who used **adenoviral vectors**. These replication-defective vectors are in theory non-integrative in most cellular types. They are able to transduce a broad range of cell types in which they remain as episomes and mediate high transgene expression according to the promoter under use [64, 65]. Stadtfeld *et al. *[63] have generated mouse iPS cells from adult hepatocytes, which correspond to adenovirus vectors best tropism. However, this process only proved successful – although at very low efficiency – when using cells which were already genetically engineered with a stably integrated inducible *Oct4 *expression cassette. More recently, with a payload of repeated infection cycles at MOI 250 with a series of adenovirus vectors expressing each a single reprogramming factor, human iPS cells could be generated from fetal fibroblasts although at much lower efficiency in reference to mouse cells [66]. When taking into account this very low efficiency, it is challenging to use adenoviral vectors with hope to generate fully reprogrammed iPS cells.

Moreover, vector and transgene integration does happen, although at low frequency. Recombination occurred overall randomly at rates between 5.5 x 10^-3^ and 1.1 x 10^-4^ but with a preference for integration into genes [67]. Altogether, at this point, adenoviral vectors might need to combine with small molecules, before being considered routinely for the derivation of human iPS cells with full stemness characteristics.

The last but not least non-integrative viral strategy that has been developed towards iPS generation, takes advantage of F-deficient **Sendai viral (SeV)** vectors. The latter replicates under the form of a negative-sense single stranded RNA in the cytoplasm of infected cells, which neither involves DNA intermediates nor may be able to integrate into the host genome [68]. Since SeV vectors are: (i) very efficient at introducing foreign genes in a wide spectrum of host cells in many species and tissues; (ii) without identified pathogenicity for human and (iii) controllable for foreign gene expression [69], they have been considered as tools for gene therapy and regenerative medicine [70, 71]. Different human somatic cell types, such as terminally differentiated circulating T cells, have been successfully reprogrammed: this is using SeV-based vectors which carry each of the reprogramming factors separately and a single infection cycle [72-76]; and the system is commercially available (DNAVEC Tsubuka). While it appears as a very appealing method, there might be limitations: for instance, the viral replicase is extremely sensitive to the nature of the transgenic sequences. In addition, because they constitutively replicate, SeV are difficult to eliminate from the host cells. However it has been shown that by passage 10, there is no residual Sendaï vector [77]. Nishimura *et al. *[78] have utmost recently reported promising results using improved SeV vectors*. *This new variant of replication defective Sendai vectors mediates persistent transgene expression (so called SeVdp), while first generation of recombinant vectors are capable of strong but transient transgene expression [79]. These SeVdp allow to generate mouse iPS more efficiently. Adding interfering RNAs to the system, SeV virus genomes could be completely eliminated. Temperature sensitive Sendai viruses have also been developed which allow drastic reduction of vector copy number in cytoplasm by a temperature shift [73, 74]. This produces iPS cells devoid of exogenous nucleic acids which translates into interesting candidates for both disease modelling and cell therapy prospects, should safety be further demonstrated.

#### 1.3.2 Transient Episomal Delivery

As an alternative to integration-defective virus, reprogramming approaches based on direct delivery of episomal vectors have been developed. These methods appear attractive since they are easy to carry out and do not require the production of viral particles. iPS cells could indeed be generated from mouse cells through both direct and transient delivery of plasmid DNA [80-82]. Si-Tayeb *et al. *[83] further addressed this option through direct delivery of plasmids otherwise used for lentivirus vectors production which encode for each reprogramming factors. Although these attempts met with some success providing two successive rounds of transfection were performed, this was at much lower rate than with lentivirus vectors. Of note, the iPS cell line generated was devoid of exogenous DNA. Along this line, Monserrat *et al. *[84] reported iPS generation from human cells in performing three consecutive cycles of transfections using the Poly(β-amino esters) polycation polymer to deliver a single polycistronic plasmid encoding for all reprogramming factors: the overall efficiency was still much lower than with virus-based systems. It may well be that fewer cells received the accurate dose of plasmids during the entire period required for reprogramming, with their premature dilution in actively dividing cells.

To circumvent the need for serial transfections and help solve the problem of episome dilution with cell divisions, Yu and colleagues [85] used **oriP/Epstein-Barr nuclear antigen-1-based episomal vectors** (oriP/EBNA1). The latter autonomously replicate as extra-chromosomal elements without integrating in the genome of cells whether dividing or not. These vectors can be maintained as stable episomes under drug selective pressure, which are progressively lost upon drug removal [86, 87]. In fact, human iPS cells were generated from human foreskin fibroblast in transfecting seven transcription factors which were expressed from three separate oriP/EBNA1 vectors. Vector- and transgene-free iPS cell lines were isolated using mere sub-cloning. However the reprogramming efficiency reported with this method proved as equally low as with other non-integrative systems [85]. More recently, two groups among which Yamanaka’s reported the generation of iPS from human dermal fibroblast and dental pulp using a combination of three oriP/EBNA1 vectors encoding for six reprogramming factors [88]. In addition, Chou *et al. *[89] obtained iPS cells from adult peripheral blood mononuclear cells by performing a single transfection round with a single polycistronic oriP/EBNA1 vector which encodes for five reprogramming factors with a 10 to 100 fold increased efficiency compared to other transient episomal delivery systems [89]*.* The later study is promising considering that patients’ peripheral blood samples are easily accessible.

Finally, in order to reduce the size of the reprogramming episomes and delete prokaryotic backbone sequences which may potentially be methylated, investigators have turned to **minicircles**. These entities represent an interesting option since they allow expression of reprogramming factors as both non-integrating and non-replicating episomes. Minicircle vectors are supercoiled DNA molecules that lack both a bacterial origin of replication and antibiotic resistance genes; therefore, they are primarily composed of an eukaryotic expression cassette. Compared to standard plasmid-DNA, minicircle vectors harbour higher transfection efficiencies and longer expression owing to decreased silencing mechanisms [90, 91] which can further be prevented by the addition of S/MARs derived sequences. A 2A-peptide-based polycistronic cassette including four reprogramming factors was used to perform several consecutive rounds of transfection which allowed Jia and colleagues [92] to generate iPS cells from human adipose stem cells with a ten-fold increase. These adipose iPS cells were devoid of vector integration. This group further published a standardized protocol for human iPS generation based on minicircle technology [93].

Significant improvements towards iPS generation have resulted from above described non-integrative strategies, but with the exception of Sendai-based vectors, all methods involve the expression of transgenes through an exogenous DNA intermediate. Although the resulting iPS cells were deemed to be transgene-free, the risk of exogenous DNA integration still persists, even at a very low rate. Therefore, careful analyses are required to scrutinize background integration and genetic stability in order to confirm that the resulting iPS cell lines are free from deleterious genetic modification.

### 1.4 Transgene-Free Delivery Methods

#### 1.4.1 RNA Delivery

Further down the road of preventing exogenous integration and suppress the risk of insertional mutagenesis, attempts have recently been made at the direct delivery of mRNAs encoding for the reprogramming factors. Plews *et al. *[94] first showed that *in vitro *transcribed capped mRNAs - which encompass 5’ and 3’ untranslated regions (UTRs) of the α-globin and encode for five reprogramming factors - resulted in increased expression of the endogenous genes responsible for cellular reprogramming. However this procedure proved insufficient to achieve full reprogramming. Few months later, Yakubov *et al. *[95] could successfully reprogram human foreskin fibroblasts by performing five consecutive transfections over several days using four *in vitro *transcribed capped mRNAs which comprise IRES sequences in the 5’UTR and a polyA signal in the 3’UTR. The best results were obtained by Warren *et al. *[96] when synthetic capped mRNAs were produced with a strong translational initiation signal in the 5’UTR and the β-globin 3’UTR with a poly-A tail signal flanking the open reading frame. As a next step of sophistication, synthetic mRNAs were protected from innate antiviral response since *in vitro *transcription was performed with 5’methylcytidine substituting for cytidine and pseudo-uridine for uridin. Repeated transfections of these synthetic mRNAs *via *cationic vehicles combined with an interferon inhibitor resulted in a conversion efficiency of about 2%; a figure which could be further increased in using chromatin structure modifiers in combination. The reprogramming efficiency achieved with this strategy is higher than with any other system, when addressing a range of human somatic donor cells under test [96, 97] and this approach is commercially available. However, the bottleneck with this method stands in the need for repeated rounds of transfection that some fragile primary cells, such as hematopoietic cells from patients with bone marrow disorders, are not able to sustain. In addition, costs related to RNA-vectors production required for repeated cycles of delivery, currently are very high (Fig. **2**).

#### 1.4.2 Protein Delivery

A last strategy which is intended at avoiding the introduction of exogenous genetic materiel into donor cells, is the delivery of reprogramming factors as proteins. A decade ago, Wilmut and colleagues showed that adult somatic cells could be reprogrammed back to an undifferentiated embryonic state using somatic cell nuclear transfer [98].

Along this line, Cho and co-workers [99] challenged cells with protein extracts derived from ES cells assuming that this could lead to similar results. Indeed, a single transfer of ES cells-derived proteins on primary cultures of mouse adult fibroblasts could fully convert iPS cells with a full differentiation potential. However, to date no human iPS cells could be generated using this approach, even when combined with chromatin remodeling small chemicals [100]. This absence of efficacy on human cells has been attributed to insufficient concentration of factors from cell extracts. In order to improve these conditions, Zhou *et al. *[101], produced recombinant reprogramming factors in *E. coli* where a poly-arginine track was fused at the C-terminus in order to facilitate their penetration across the plasma membrane [102]. Following four cycles of exposure to the purified recombinant proteins and the concomitant addition of a HDAC inhibitor, iPS cells were isolated from MEFs. However, again, so far attempts to establish human iPS cells using this method have been unsuccessful. In addition, substantially large amounts of purified recombinant proteins are required which make it unlikely to be tailored for routine use. The same year, another group was luckier starting from the human HEK293 cell line engineered as a donor source to stably express one recombinant reprogramming factor also fused to a poly-arginine track. Human neonatal fibroblasts were exposed to protein extracts derived from the HEK293 cell line at regularly intervals, consisting of consecutive cycles of eight hours per week during six weeks, after which few iPS colonies could be isolated [103]. These protein-based strategies might be relevant when considering that iPS cell lines are completely devoid of exogenous DNA, thereby suppressing the risk for insertional mutagenesis which stems from integration of foreign DNA sequences into the genome. While poor efficiencies would require improvement, the genuine prevention of genomic instability also needs to be demonstrated when considering in particular the extremely slow kinetics of the induction process based on proteins delivery.

## 2 REPROGRAMMING

Direct reprogramming is conceptually simple which involves ectopic introduction of defined factors that are capable of inducing cell conversion: the related induction technologies currently are widely used in many laboratories. However, this process still is extremely slow, inefficient, and depends on several parameters which affect efficiency, reproducibility in the process and the quality of the resulting iPS cells. As discussed in the previous section, one parameter is related to the method selected for reprogramming since virus-based systems are more efficient than the transfer of naked nucleic acids or the direct addition of proteins for example. However, the precise selection of those reprogramming factors that will be used in accordance with the donor cell types, also is a key element of success and/or safety which is discussed in the next section (Table **2**).

### 2.1 Reprogramming Factors to Facilitate Stem Cells Induction

#### 2.1.1 “Conventional” Cocktails

Since Yamanaka’s group reported the generation of mouse and human iPS cells via retroviral-mediated ectopic expression of ***OCT4*** (also known as *POU5F1*), ***SOX2***, ***KLF4*** and ***c-MYC*** (so called OSKM cocktail) [5, 104], this canonical cocktail now has proved efficient on a wide range of human cell types with integrative delivery systems, in particular, as recently reviewed by Gonzalez *et al. *[17]. The **OSKM** cocktail was also shown to perform when introduced with non-integrative systems such as Sendai viruses [72, 75, 76, 78] or mRNAs [96], and although at very low efficiency in particular with adenoviruses [101], episomal plasmids [84], and proteins [103].

As early as one month after the publication of Yamanaka’s work on human cells, Thomson and colleagues reported the generation of human iPS using another reprogramming cocktail which also comprises *OCT4 *and *SOX2*, and involves ***NANOG*** and ***LIN28*** (**OSNL**) instead of *KLF4 *and *c-MYC *[13]. This reprogramming cocktail has also proved efficient in most cases when delivered by lentiviruses [17] or as mRNAs [95]. The stoichiometry of the reprogramming factors has been investigated: Papapetrou *et al. * [105] have shown that a high expression of *OCT4*, compared to others factors, is required with view to increasing conversion. When moving to polycistronic vectors, the main factor conditioning success is a high transduction efficacy. Cocktails including five factors such as OSKMN or OSKNL have further been tested in order to either improve the efficiency of iPS cells generation from common cell types such as keratinocytes and fibroblasts [33, 36] or facilitate the reprogramming of more difficult cells such as diseased patients’ cell and vascular smooth muscle cells [16, 106]. The simultaneous use of six-reprogramming factors (OSKMNL) has further been attempted which met with additional success with human new born foreskin and fetal dermis fibroblasts [107, 108]. A variety of other pluripotency-related factors have also been tested such as *UTF1 *[109] with which more colonies where obtained when expressed along with OSKM in human primary fibroblasts. Similarly, a ten-fold increase could be observed when *SALL4 *was co-expressed with OSK in human adult fibroblasts from dermis [110] (Tables **1** and **2**).

#### 2.1.2 Reprogramming Efficacy is Tightly Linked to Cell Proliferation

The efficiency with which cells can be converted is directly linked to cell cycle and division status. Indeed, a high proliferation rate appears to be required for efficient cell reprogramming [111]. As a consequence, when combined with OSKM both the SV40 large T antigen (SV40LT) and the Telomerase reverse transcriptase (h*TERT*) known to have positive effects on cell proliferation and prevention of cell senescence in protecting chromosome ends, increase the number of iPS colonies [16]. Along the same rationale, *REM2 *or *CyclinD1 *expression enhances reprogramming compared to the “conventional” cocktail alone and more importantly allows iPS generation without involving *c-MYC *[112]. As SV40LT is known to target p53, it has been hypothesized that p53 inhibition could also behave as a facilitator. Several studies have reported that the use of short hairpin RNAs (shRNAs) against p53 does indeed enhance cell conversion efficiency [109, 113, 114]. Further studies have defined p53 as a guardian against reprogramming [115] as i) p53-p21 pathway prevents iPS cells generation [116, 117] and ii) during the reprogramming process, the levels of both p53 and p53 targets are increased and iii) p53 induces growth arrest and apoptosis [104, 105, 107]. Although Mah *et al.* [118] have postulated that these observations correspond to the innate immunity response induced by viral transduction, Hong *et al. *[116] reported that this response may indeed appear to be independent of viral integration. In further experiments, the introduction of shRNAs against p53 allowed iPS generation in the absence of *c-MYC* (OSK cocktail) as well as in the absence of *KLF4 *(OS cocktail) on keratinocytes as shown by Kawamura *et al. *[113]. In postnatal neurons (although post-mitotic), the addition of a short hairpin RNAs (shRNA) against p53 to the OSKM cocktail is compulsory to successful reprogramming [119]. Another roadblock that is limiting reprogramming efficiency is the *Ink4a/ARF *locus which is linked to the p53 pathway. Indeed, shRNAs against *ARF *and/or *Ink4a *have been shown to greatly improve cell conversion efficiency in the absence of *c-MYC *in fetal lung fibroblasts [120].

**The obvious influence of cell-cycle regulators** has also been evidenced using small chemical molecules. Indeed, the inhibition of either or both the mitogen-activated protein kinase kinase (known as MEKK) signalling and Glycogene Synthase Kinase 3 (GSK3) pathways increases the number of fully reprogrammed colonies [83, 121]; in addition, this allows full reprogramming of neural precursors without a requirement for SOX2 and c-MYC [122]. Finally, MEK inhibitors promote the transformation of fibroblasts into stem cells with a 200-fold increase over the classical method in combination with an ALK5 (TGFβ receptor) inhibitor and thiazovivin [123]. While playing with identified key regulators of the cell-cycle clearly results in the facilitation of adults cell conversion into iPS, scrutiny is required on the potential associated payload when genetic stability and controlled proliferation might be at stake.

**Cell cycle rate is directly linked to cell cycle checkpoint.** Whereas somatic cell primarily use non-homologous end joining (NHEJ) DNA repair mechanism, pluripotent cells mainly rely on homologous recombination (HR) DNA repair pathway to maintain genomic integrity. DNA damage response is activated during reprogramming process [124] which is correlated to the accumulation of γH2AX. Consistently, the reprogramming efficiency decreased dramatically in p53BP1 and ATM deficient cells [114]. Another study demonstrated that defects in the Fanconi anemia (FA) DNA repair pathway led to poor reprogramming efficiency which could be restored by ectopic expression of FANCA in *Fanca* -/- cells [121, 125]. These observations indicate the important roles of DNA damage repair pathways in reprogramming.

**Specific microRNAs (miRNA)** have been shown to be involved in pluripotency and reprogramming [126] such as the miR-290 cluster which is believed to act downstream of *MYC *and is involved in features unique to EC cell-cycle [127]. The use of miR-291-3p, miR-294 or miR-295 combined with *OSK *cocktail increases the reprogramming efficiency in MEFs [128]. However, to date no human iPS generation has been reported using these miRNAs. Finally, recent studies have evidenced that both Oct4 and Sox2 play a pivotal role in miR-302 expression in human embryonic stem cells (hES) [129, 130]. MiR-302 indeed belongs to a class of miRNAs that functions as cytoplasmic gene silencers: this is in suppressing translation of targeted messenger RNAs (mRNA). A majority of miR-302-targeted genes are transcripts involved in development-related signals and oncogenes [131]. In human, miR-302 is predominantly expressed in hES and iPS cells, but not in differentiated cells [132, 133]. In using a vector which expresses a cDNA encoding for miR-302 and further selecting cells for its stable integration with antibiotics, Lin and co-workers [30] were able to achieve full reprogramming of cells from human hair follicles; however that cells are slow to propagate because of a restricted cell cycle rate [134].

**Culture conditions** can also modulate reprogramming efficiencies as a four-fold increase in human cell conversion efficiency is observed when MEFs are maintained under 5% O_2_ hypoxic condition (like in stem-cell niches) during the reprogramming process which allows iPS generation with only two factors OCT4 and KLF4 [135]. This data is in keeping with well-identified observations of improved survival of hematopoietic stem cells [136] and the prevention of human ESCs differentiation [137] under low O_2_ tension. In fact, pluripotency is regulated by the family of hypoxia inducible factors (HIFs) among which [HIF-2α] has been shown to act as an upstream regulator of OCT4 which in turn is also involved in both NANOG and SOX2 expression [138].

#### 2.1.3 Overcoming Epigenetic Barriers

iPS reprogramming overall is a rather inefficient process. Somatic cell conversion obviously involves a massive reconfiguration of the chromatin structure, from DNA methylation to histone and nucleosome modifications. Chromatin remodelling, also known as the epigenetic barrier, is a rate-limiting step in somatic cell reprogramming since it holds the power to abrogate unwanted expression of lineage specific genes. The added value of chemical compounds which can modulate either DNA methylation status or chromatin modifications have emphasized the importance of epigenome in reprogramming. Subsequent improvement has been evidenced in various cell types.

For example, the inhibition of DNA methylation during the conversion phase with the DNA methyltransferase (DNMT) inhibitor 5-azacytidine allows mouse iPSCs which exhibit an intermediate pattern to be fully reprogrammed [139]. Vitamin C also significantly improves reprogramming efficiency as it alleviates cell senescence [140] and induces DNA demethylation of gene sets specific to cell conversion [141]. Treatment with histone deacetylase (HDAC) inhibitors such as trichostatin A (TSA) or valproic acid (VPA), induces chromatin remodelling leading to up-regulation of ESC-specific genes [123], improvement of somatic cell reprogramming efficiency and also allows cell conversion with only two factors: *OCT4 *and *SOX2 *[31]. Upon addition of VPA, cell conversion could be demonstrated *via *the direct delivery of recombinant proteins (OSKM cocktail) in mouse cells [101]. Picanço-Castro and colleagues have generated iPS cells from human dermal fibroblasts in combining VPA with viral delivery of c-MYC, Sox2 and TCL1-A [142], a co-activator of the cell survival kinase AKT [143]. Recently, c-KIT+ amniotic fluid stem cells could be fully reprogrammed to pluripotency without any ectopic factors by culture in hESC medium supplemented with VPA [144]. Butyrate also affects both histone H3 acetylation and promoter DNA methylation, thus altering the expression of endogenous pluripotency associated genes. As a consequence, it is expected to greatly enhance iPS cell derivation from human adult or fetal fibroblasts using 4 to 5 reprogramming genes; furthermore, its effect on reprogramming is more remarkable with an increase by over a 100- to 200-fold in the absence of either *KLF4 *or MYC [145]. Along the same line, by inhibiting G9a histone methyltransferase, which mediates epigenetic repression of *OCT4 *[146], iPS could be generated from MEFs using only two factors: *OCT4 *and *KLF4 *[32]. Other authors have also used Tranylcypromine (Parnate), an inhibitor of lysine-specific demethylase 1, which is responsible for K4 demethylation. They could successfully generate iPS cells from human keratinocytes again with *OCT4 *and *KLF4 *only [123]. However, chemical compounds could have deleterious side effects. For example, VPA has been shown to enhance recombination events [147, 148] and reduce the ability of cells to repair DNA double-strand breaks [149]. It might thus be wise to weigh out the use of these DNA-modifying molecules when considering potential consequences of their use on the genetic stability of resulting iPS cells.

### 2.2 Bottlenecks Towards Clinical Translation

#### 2.2.1 Preventing the Risk for Induced Oncogenesis

**Addressing the nature of reprogramming factors**: In addition to the risk for insertional mutagenesis related to integration of foreign sequences into the cell genome, a forced expression of reprogramming factors may bring along an additional risk for the development of malignancy, when considering both the nature and the combination of inducing factors under use. In fact, among proposed procedures some are reminiscent of the generation of immortalized cell lines such as ectopic expression of telomerase reverse transcriptase (h*TERT*) and/or SV40 large T antigen (SV40LT) and their propensity for malignant transformation, *e.g*.: by adding a single oncogene such as H-ras [150-152]. Therefore, the potential added value to the reprogramming process related to the addition of these factors should be carefully weighed out in the eye of their potential incompatibility with clinical-relevant prospects. Similarly, the inclusion of the c-Myc proto-oncogene is also controversial as it is associated with tumour formation in iPSC-derived chimeric mice [5], despite a well-identified potent promoter of iPSC generation. This promotion capacity nevertheless is independent from its transformation property; indeed, other members of the Myc family such as L-Myc, or mutant c-Myc, share this ability to promote iPSC generation while showing more specific and efficient as compared to WT c-Myc [88, 153].

**Reducing the number of reprogramming factors:** since the most efficient and commonly used methods to induce adult cell conversion involve the stable integration of transgenes with the concurrent risk for insertional mutagenesis, a critical path for improvement consists in the reduction of transduced reprogramming factors. So far, fibroblasts - which remain the most common donor cell type used in over 80% iPS experiments published so far [17] - were successfully reprogrammed using three-factors cocktails: whether including OSK [23, 41, 154-157], OSM [155] or OSN [158]; though at lower efficiency. Human keratinocytes, as well as mesenchymal cells from teeth and dental pulp have also been converted with OSK [19, 159, 160]. The endogenous expression of at least one of the reprogramming factors in some cell types obviously facilitates their full reprogramming. For instance, amniotic derived cells which spontaneously exhibit robust expression of *c-MYC*, could be converted with three-factors cocktails like either OSK [123, 161, 162] or OSN [163]. Along the same line, human melanocytes were found to express Sox2 and were reprogrammed with the OKM cocktail [37]. The challenge of cell conversion with the introduction of the two factors *OCT4 *and *SOX2 *only, met with success in human endothelial cells from umbilical cells that harbour endogenous expression of *KLF4 *[164]. Similarly, fetal neural stem cells, which express high level of endogenous *SOX2, *could be converted using *OCT4 *and *KLF4 *[165] and further *via *ectopic expression of *OCT4 *only [166]. However, these immature cells are relatively inaccessible and difficult to obtain and cannot be considered as straightforward sources for routine use. Such limitations might nevertheless be overcome: Giorgetti *et al. *reported promising results from studies involving CD133+ cells from cord blood which only require expression of *OCT4 *and *SOX2 *to convert into iPS cells [167]. As with other applications, including allogenic blood stem cells transplantation, these cells which may be available from cell banks and are easy to isolate offer significant advantages over other adult somatic cell sources (Table **2**).

#### 2.2.2 Donor Cell Type and Differentiation Efficiency

Embryonic tissues are the most easily prone to reprogramming, a process which results in this particular case in iPSCs which are nearly identical to fetal ESCs (fESCs). In contrast, reprogramming from commonly accessible adult tissues, which hold the utmost potential for disease modelling, is less efficient since it is limited by barriers related to donor’s cells age and differentiation status [114, 120, 168]. Ageing cells harbour higher levels of Ink4/Arf, which limits the efficiency and fidelity of the reprogramming process [120].

Similarly, terminally differentiated blood cells can less efficiently be converted when compared to blood progenitors [168]. As mentioned above, various adult tissues show uneven susceptibility to reprogramming and reprogramming efficiency seems to vary depending on methods and laboratories [36, 169]. Interestingly, iPSCs from stomach or liver cells harbour fewer integrated proviruses than fibroblasts, a feature which might indicate that lower expression levels of reprogramming factors may be required to achieve pluripotency [170]. Of note, cells may sit in intermediate states of reprogramming (so-called “interconvert”) and achieve full conversion through sustained passages or treatment with chromatin-modifying agents [139, 171].

Fully reprogrammed generic iPSCs are highly similar to fESCs: like fESCs, iPSCs form teratomas *i.e.*: differentiated benign tumours which involve tissues from all three embryonic germ layers (Table **3**). Nevertheless both functional and molecular significant differences may be evidenced in iPSCs generated from various tissues. Human iPS cells have been suggested to be less prone to differentiation into either neural or blood tissue lineages [172, 173]. Since reversion of methylation is identified as a slow and inefficient process, it has been postulated that residual methylation remains within iPSCs. It was indeed recently shown that both mouse and human iPS cells exhibit noticeable variability in their epigenome. Genome-wide studies have revealed that although being close to ES cells [174-178], iPSC harbour differentially methylated regions (DMRs), which also vary from one line to another [175, 179, 180]. This particular feature also is associated with reprogramming variability [174, 181, 182]. In theory, the reprogramming process would likely erase all tissue specific marks; however, iPS cells do harbour DMRs which are hallmarks of the three-germ layers and of normal development status [175]. In most cases, different epigenetic features observed between iPS cells are characteristic of the tissue from which they originate which is defined as **epigenetic memory** [174-177]. In addition, it has been further shown that, at low passage, iPS cells retain persistent expression of somatic genes. This **transcriptional memory** is believed to result from both incomplete silencing of tissue specific genes and potentially incomplete reactivation of ES cell specific genes during the reprogramming process, a phenomenon which might partially be explained by promoters incomplete DNA methylation [178]. Residual epigenetic marks in fact antagonise differentiation into cell lineages distinct from the donor cell type and restrict the downstream process to the latter [175-177]. In studies performed with murine iPS cell lines, this epigenetic memory can be erased over time by extended culture [183]. Nevertheless this observation does not hold true in human iPS cells although cells show a gradual increase in their differentiation potential [177]. Interestingly, several rounds of reprogramming may expand iPS cell differentiation potential towards additional lineages as property to expand the differentiation potential shown by Kim *et al.* [176].

Finally, the does not necessarily seem to correlate with the age of the donor. Along the same line of investigation, neither significant difference were identified between iPS cell lines originating from healthy *versus* diseased patients nor between lines reprogrammed with three *versus *four-factors [181]. Furthermore, the impact of reprogramming factors stable integration is controversial: Soldner *et al. *[42] claim that only viral excision can resolve the bottleneck of gene expression signature which is observed in differentiated progeny of iPS cells; on the other hand, Boulting *et al. *[181] were not able to detect any effect on differentiation in cell which display persistent transgene expression.

#### 2.2.3 Is Genetic Instability a Payload to Reprogramming?

As previously mentioned, like ES cells, iPS cells exhibit variability in their epigenetic, transcriptional and differentiation potential, which in most case, represent a somatic memory originating from features specific to the donor cell. Bock and co-workers postulate that somatic cell memory provides a potential explanation to some iPS deviation although this phenomenon involves a small fraction of overall differences observed in the DNA methylation and gene expression profiles observed in iPS cell lines [174].

Aberrant epigenetic profiles were reported in several studies [175, 182, 184]. It thus appears that iPSC lines which were generated in various laboratories, using distinct technologies and derived from different germ layers, share numerous non-randomly distributed megabase-scale regions that are aberrantly methylated in a non-GC rich context. They are associated with alterations in CG methylation, histone modifications and gene expression. Moreover, the somatic reprogramming efficiency of somatic cell lines is inversely correlated to the amount of methylation change needed to acquire pluripotency. However, a specific reprogramming-associated epigenetic signature has been identified, which allows to segregate between hESC and hiPSC lines [184]. These DMRs observed in iPSCs are actually transmitted to their differentiated progeny at high rate [182, 184]. 

When considering more subtle modifications, hiPS cell lines have been shown to contain an average of five protein-coding point mutations in the regions sampled (six protein-coding point mutations per exome estimate): this observation is concordant in cell-lines which had been derived by means of five different reprogramming methods. Most of these mutations were non-synonymous, nonsense or splice variants, and were enriched in genes mutated or having well-established causative effect in cancers. Of note, at least half of these reprogramming-associated mutations pre-existed in fact yet at low frequency in the fibroblast progenitors, the remaining half undoubtedly occurred during or after reprogramming [185]. When turning to copy number variations (CNVs) analysis through high-resolution nucleotide polymorphism array, significantly more CNVs are present in early-passage human iPS cells *versus *intermediate passage. Most CNVs are formed *de novo *and generate genetic mosaicism. Hussein and co-workers show evidence that the process of human iPS cells expansion in culture rapidly selects against affected mutated cells: this self-resolving dynamic subsequently drives the iPS line toward a genetic state resembling human ES cells [186]. Whereas experience gathered does validate the hypothesis that massive genetic alteration resolve in cell-catastrophe following death signals, more subtle genetic alterations can conversely accumulate and select in favour of mutated clones with selective growth advantage: in the X-SCiDs gene therapy clinical trials performed in both Paris and London, three years were required in patients *in vivo *for malignant clone outgrowth resulting in leukemia [187, 188].

These studies clearly indicate that both the reprogramming process and the subsequent expansion of iPSCs in culture may carry along the accumulation of genetic abnormalities at the chromosomal, sub-chromosomal and single-base levels. Hussein *et al. *[186] provide evidence that CNVs occurred more frequently at sites prone to replication stress. This observation suggests that the reprogramming process and the strong selection which is associated thereof, generate huge pressures on DNA replication and cell growth, which in turn result in genetic aberrations. Moreover, genetic amplification, deletion or point mutation lesions that arise in iPSCs mostly involve regions prone to cell-cycle regulation and cancer [185, 189, 190]. Although observed modifications during the amplification of iPSCs or their adaptation to culture conditions do not target a specific gene, the frequent association of genes affected with cancer gives cause for concern. This question is still in balance since it has recently been shown that reprogramming does not necessarily lead to de novo CNVs in iPSCs since some modifications have been evidenced as being already present as somatic genomic variants in parental fibroblasts [191]. This raises the issue of inducing iPS from skin fibroblasts which have commonly been exposed to UV as the most used cell-type for reprogramming so far. Indeed, such observations might disqualify skin fibroblasts as the best source-candidate with view to future prospects of clinical translation. Ensuring cell safety and genome integrity of hiPS through extensive genetic screening should therefore become a standard procedure before any clinical use would be considered at all. 

## CONCLUSION

iPSCs represent a widely available, non-controversial and practically infinite source of pluripotent cells. Unlike human ESCs, their use is not restricted for ethical reasons, allowing most laboratories to develop research programmes involving this source of human pluripotent stem-cell lines. Since the first published demonstration from Yamanaka’s laboratory that fibroblasts can be reprogrammed merely by retroviral delivery of four factors (OSKM), many alternative approaches have been developed in order to induce pluripotency starting from adult somatic cells. Integrative strategies based on retrovirus or transposons mediated gene transfer are most efficient and can be used for prominent current applications such as disease modelling and therapeutic screens, since the absence of persisting genetic modification is not an absolute prerequisite. In contrast, the generation of clinically relevant iPSCs intended for future cell therapy prospects requires technological approaches which do not leave genetic traces behind the cell conversion phase. Although methods based on proteins delivery [99, 101, 176] are relatively inefficient, strategies involving RNAs, directly or *via *Sendaï virus, and their potential improvement seem promising owing to the high efficiency of cell reprogramming [72, 76, 96].

However, ‘safer’ approaches without genetic scars, do not necessarily prevent variability in lineage-specific genes expression levels or the occurrence of aberrant epigenetic remodelling. Consequently, a pivotal challenge in the iPSC field is to determine how various methodologies affect the quality and the genomic integrity of iPSCs. Whole-genome sequencing and epigenome screening will probably play an important part in the validation of the iPS cell lines generated in terms of transcriptional signatures, epigenic status, genomic integrity, stability, differentiation and tumour potential.

Prospects for human iPSCs-based cell therapies have been considered which raise enthusiasm toward regenerative medicine application and tissue-replacement to treat injuries or diseases; iPSCs could in theory be generated in an autologous context. Another exciting application of hiPSC is to constitute a cell bank of allogenic hiPSC readily available to cover most histocompatibility complex combinations worldwide, intended for cell transplantation, so called “Haplobank” [192]. Beside a requirement for improved induction strategies and validation methodologies to ultimately warrant safety, iPSCs-based cell therapies will also require in many instances, the correction of genetic defects. Recently, the development of the “Zinc Finger Nuclease” (ZFN) technology enables efficient and precise genetic modifications *via *the induction of a double-strand break in a specific genomic target sequence, followed by the generation of desired modifications during subsequent DNA repair. This process is allowed as ZFN architecture links a DNA-binding domain of eukaryotic transcription factors customised to cleave a specific DNA target sequence and the nuclease domain of the FokI restriction enzyme [193-196]**.** Li *et al. *[197] recently achieved *in vivo *genetic correction of haemophilia B *via *ZFN genome targeting and shown persistent correction. Although the relative efficiency of gene targeting still remains under the 1% range, clinical translation of ZFN gene targeting is currently underway in three Phase I clinical trial for the treatment of glioblastoma [198] and HIV [199, 200]. Recently, Yusa and co-workers [201] achieved biallelic correction of a point mutation in the gene *A1AT *responsible for a1-antitrypsin deficiency in diseased iPSCs, using a combination of ZFN and Piggy-Bac technologies, which restores both the structure and function of A1AT in liver cells derived *in vitro *and *in vivo*. Finally, new site-specific nucleases have been developed through engineering of Meganucleases and Transcription Activator-Like Effectors, so called TALENs. These nucleases mediate site-specific genome modifications in human pluripotent cells with similar efficiency and precision as do zinc-finger nucleases [195, 196, 202] and with far less toxicity, as reported. Once combining safe iPSCs induction and homologous recombination will become available, autologous cell-based therapies might be within reach providing clinically relevant cells can be established. This reality is not so far since very recently a therapeutic gene could be inserted in place of the reprogramming cassette in combining both technologies [203]. 

## Figures and Tables

**Table 1.** This Table Tentatively Recapitulates Delivery Methods and Combination of Technological Options Used for iPS Induction, Starting from Delivery System as Follows: Transgenes Under Use ; Addition of Chemicals and Cell-Type Converted. The PMIDs of Key Papers Describing the Methods and its Outcome are Listed in the Last Column, in the Corresponding Line

Delivery Method		Transgene	Chemicals	Cell type	PMID

**Integrating Vectors**	Retrovirus	OSKM	-	Fibroblast-like synoviocytes	18035408
				Fibroblasts, fibroblasts (primary: BJ)	

	Polycistronic retrovirus	OSKM/OSK	-	Fibroblasts (foreskin, xeno-free primary culture)	19890879

	Lentivirus	OSLN	-	Fibroblasts (IMR90)	18029452
				Fibroblasts (neonatal foreskin)	

	Inducible Lentivirus	OSKM/OSK	-	Bone marrow mesenchymal stem cells (diseased patients), fibroblasts (diseased patients)	18691744

		OSKM	-	Fibroblasts (fetal dermal)	20572011
				Fibroblasts (fetal lung)	

		OSKMN/OSKM	-	Fibroblasts (differentiated from iPS), Fibroblasts (neonatal foreskin), keratinocytes	18786420

		OSKM	-	Peripheral blood myeloid cells peripheral blood T cells	20621045

		OSKMN		Fibroblasts	20569691
				Fibroblasts (secondary)	

	Polycistronic lentivirus	OSKM	-	Fibroblasts	20682452

	Inducible polycistronic lentivirus	OSKM	-	Keratinocytes (foreskin)	19109433

	Inducible Plasmid	mir-302	-	Hair follicle cells	20870751

**Excisable integrating vector**	Excisable (LoxP) lentivirus	OSKM/OSK	-	Fibroblasts (skin of patients suffering from Parkinson disease)	19269371

	Excisable (LoxP) polycistronic lentivirus	OSK	-	Fibroblasts (adult humanized sickle cell anemia mouse)	19415770

	Excisable (FRT) polycistronic lentivirus	OSKM	-	Murine fibroblasts SC1	20385817

	PiggyBack Transposon	OSKM	-	Fibroblasts (embryonic)	19252477

			Butyrate	Fibroblasts (fetal lung, IMR90)	18511599

	Inducible PiggyBack Transposon	OSKM	-	Fibroblasts (embryonic)	19252478

	Sleeping Beauty	OSKM	-		Izsvak *et al.* 2011

**Non-integrating vectors / pathways**	Adenovirus	OSKM	-	Fibroblasts (IMR90)	19697349

	Sendaï virus	OSKM	-	Neonatal foreskin fibroblasts BJ Dermal fibroblasts	19838014

		OSKM	-	Terminally differentiated circulating T cell	20621043

	Lentivector (plasmid)	OSLN	MEK inhibitor	Fibroblasts (foreskin)	20682060

	EBV based plasmid	OSKMNL + TSV40 + shRNAp53	-	Fibroblasts (neonatal foreskin)	19325077

		OSKMNL + TSV40	-	Neonatal cord blood, adult peripheral blood mononuclear cells	21243013

		OSKL+L-Myc+ shRNAp53	-	Dermal fibroblasts, Dental pulp cell line	21460823

	Polycistronic plasmid	OSKM	poly(β-amino esters)	Fibroblasts (foreskin)	21285354

	Minicircles	OSLN	-	Adipose stem cells	20139967/ 21212777

	RNA	OSLN	-	Fibroblasts (foreskin)	20188704

		OSKML/OSKM	-	Fibroblasts (fetal lung), fibroblasts (fetal skin), Fibroblasts (foreskin)	20888316
				Fibroblasts (neonatal foreskin)	
				Fibroblasts (skin from cystic fibrosis patient)	

	Proteins	ES cell extracts	-	Mouse fibroblasts: primary cardiac and primary skin	20439621

		OSK/OSKM	VPA	Mouse fibroblasts	19398399

		OSKM		Neonatal fibroblasts	19481515

Abbreviations: O, *OCT4*; S,*SOX2*; M, *c-MYC*; L,*LIN28*; N, *NANOG*; TCL-1A, T-cell leukemia/lymphoma protein 1A.

**Table 2.** This Table – Complementing Table 1 – Comprehensively Describes the Factors/ Transgenes and Chemicals Used in Order to Achieve Cell-Conversion. The Following Columns Provide Details on the Matching Delivery Methods Under Use and in which Cell-Type. The PMIDs of Key Papers are Listed in the Last Column, in the Corresponding Line

Transgenes		Chemicals	Delivery method	Cell type	PMID

**Original**	OSKM	-	Retrovirus	Fibroblast-like synoviocytes	18035408
				Fibroblasts, fibroblasts (primary: BJ)	

	OSLN	-	Lentivirus	Fibroblasts (IMR90)	18029452
				Fibroblasts (neonatal foreskin)	

**4 factors or more**	OSKMNL + T SV40 + shRNAp53	-	EBV based plasmid	Fibroblasts (neonatal foreskin)	19325077

	OSKMNL + T SV40	-	EBV based plasmid	Neonatal cord blood, adult peripheral blood mononuclear cells	21243013

	OSKMNL	-	Lentivirus	Fibroblasts (neonatal foreskin)	18414447

		-	Lentivirus	Fibroblasts (dermal)	20524893

		-	Lentivirus/Retrovirus	Fibroblasts	20354136

	OSKL+L-Myc+ shRNAp53	-	EBV based plasmid	Dermal fibroblasts, Dental pulp cell line	21460823

	OSKMN	-	Inducible lentivirus	Fibroblasts (differentiated from iPS)	18786420
				Fibroblasts (neonatal foreskin)	
				Keratinocytes	

		-	Inducible lentivirus	Fibroblasts fibroblasts (secondary)	20569691

		-	Inducible lentivirus	Bone marrow mesenchymal stem cells (diseased patients), Fibroblasts (diseased patients)	18691744

	OSKML	Butyrate	PiggyBac	Mesenchymal stem cells	18511599

	OSKNL	-	Lentivirus	Aortic vascular smooth muscle cells	19959777

	OSKM + *UTF1*/ OSKM + *UTF1*+shRNAp53	-	Lentivirus	Fibroblasts (adult foreskin) fibroblasts (fetal skin)	18983962

	OSKM + *hTERT*/ OSKM + T SV40	-	Inducible lentivirus	Bone marrow mesenchymal stem cells (diseased patients), Fibroblasts (diseased patients)	18691744

	OSKM + shRNAp53	-	Retrovirus	Fibroblasts (primary: BJ)	19668189

		-	Retrovirus	Postnatal neurons	21563275

	OSKM	MEK inhibitor + GSK3 inhibitor	Retrovirus	Disease-corrected fibroblasts (dermis of Fanconi anaemia patients), Disease-corrected keratinocytes (epidermis of Fanconi anaemia patients), Fibroblasts (foreskin)	19483674

		Butyrate	Retrovirus	Fibroblasts (fetal lung, IMR90)	18511599

	OSK+L-*Myc*/ OSK+n-*Myc*	-	Retrovirus	Fibroblasts (dermal)	20660764

	OSNL OSNL	MEK inhibitor	Lentivector (plasmid)	Fibroblasts (foreskin)	20682060

		MEK inhibitor + GSK3 inhibitor + TGFbR1 inhibitor	Lentivirus	Fibroblasts (IMR90)	19097958

	OSK + *SALL4*	-	Retrovirus	Fibroblasts (adult dermis)	19476507

	OSK + *REM2*/ OSK + *CycD1*	-	Polycistronic retrovirus	Keratinocytes (foreskin)	20231315

	OSK + shRNA ARF/Ink4a	-	Retrovirus	Fibroblasts (IMR90-TERT)	19668188

**3-factors reprogramming cocktails**	OSK	Vitamin C	Retrovirus	Adipose stem cells, periosteal membrane cells, placental corionic mesenchymal cells, skin fibroblasts from fetus with beta thalassemia	20036631

	OSK	-	Retrovirus	Fibroblasts (adult dermis)	18059259

		-	Retrovirus	Fibroblasts (adult dermis), fibroblasts (fetal lung), Fibroblasts (foreskin)	19688839

			Retrovirus	Fibroblasts (foreskin, xeno-free primary culture)	19890879

		-	Retrovirus	Fibroblasts (neonatal foreskin)	20861676

		-	Inducible retrovirus	Fibroblasts, fibroblasts (differentiated from iPS)	18786421

		-	Retrovirus	Hair (single plucked), keratinocytes (foreskin)	18931654

		-	Retrovirus	Dental pulp cells	20554890

		-	Retrovirus	Mesenchymal stromal cells (from human third molar)	20595386

		-	Retrovirus	Extra-embryonic amnion cells	19912344

		-	Retrovirus	Amniotic fluid cells Chorionic villus sample	19482945

		-	Retrovirus	Amniotic fuild-derived cells (hAFDCs)	19679563

		-	Retrovrus	ES-derived fibroblasts	18287077

	OSM	-	Retrovrus	ES-derived fibroblasts	18287077

	OSN	-	Lentivirus	Fibroblasts (adult dermis)	19259936

		-	Lentivirus	Amnion derived cells (hADC)	20510497

	OKM	-	Inducible lentivirus	Melanocytes	19723802

	OS + shRNAp53	-	Retrovirus	Fibroblasts (IMR90) Keratinocytes	19668186

**2-factors cocktails**	OS	VPA	Retrovirus	Primary fibroblasts : BJ, NHDF	18849973

		-	Retrovirus	CD133+ cord blood stem cells	19796614

		-	Lentivirus	Umbilical vein endothelial cells	20689077

	OK	GSK3 inhibitor + Parnate	Lentivirus	Keratinocytes (epidermal)	19830055

		-	Retrovirus	Neural stem cell (embryonic)	19763260

	MS + *TCL-1A*	VPA	Lentivirus	Fibroblasts (adult dermis)	20504151

**1F**	O	-	Retrovirus	Neural stem cells (fetal)	19718018

	mir-302	-	Inducible Plasmid	Hair follicle cells	20870751

No Factor		VPA	None (chemical only)	Amniotic fluid Stem Cell	23050522

Abbreviations : O, *OCT4*; S,*SOX2*; M, *c-MYC*; L,*LIN28*; N, *NANOG*; TCL-1A, T-cell leukemia/lymphoma protein 1A.

**Table 3.** Methods and Markers Aimed to Characterize Fully Reprogrammed iPS

Detection methods	Markers

Immunostainning	Alkine Phosphatase
	**OCT4, NANOG, SOX2, KLF4, **Tra-1-60, **Tra-1-81**, c-MYC, SSEA1, **SSEA3, SSEA4**

Flow cytometry	**OCT4, NANOG, SOX2, KLF4, **Tra-1-60, Tra-1-81, c-MYC, **SSEA3, SSEA4**

Western Blotting	**OCT4, SOX2, KLF4,** c-MYC

qRT-PCR	Endogenous ***OCT4, SOX2, KLF4,NANOG, LIN28, hTERT, REX1****, SALL4,DPPA2, DPPA4 GDF3, cMYC, PPIA, DNMT3B* should be re-activated;
	Exogenous reprogramming transgenes should be inactivated;

Bisulfite sequencing	Endogenous* OCT4, NANOG* promoter regions should be activated*; *
	Promoter regions driving exogenous reprogramming transgenes expression should be inactivated;

Teratoma formation in immuno-deficient mice	Differentiation in various cell types from all three germ layers

Embryoid body formation	

In this table, methods to identify the accurate markers are listed in order to determine whether a fully reprogrammed iPS cell line has been established.
